# What we could not have known: ovarian cycles, follicle development, and the signature of life

**DOI:** 10.1093/molehr/gaag010

**Published:** 2026-03-05

**Authors:** Teresa K Woodruff

**Affiliations:** Department of Obstetrics and Gynecology, Michigan State University, East Lansing, MI, USA; Department of Biomedical Engineering, Michigan State University, East Lansing, MI, USA

**Keywords:** inhibin, activin, oncofertility, zinc spark, follicle, EVATAR, microfluidics, *in vitro* follicle growth

## Abstract

Reproductive science is a discipline of generation and for generations. Our work has focused on the signals that drive reproductive cycles at the organismal and the organ level. The principal endocrine signals of male and female reproductive cycles include inhibin and activin, traditional peptide hormones, and at the cellular level, zinc, a workhorse element now elevated to the role of a signal on par with phosphate and calcium. Our studies relied on and built technologies to make our discoveries, including enzymatic sequencing and PCR, alginate beads and microfluidics, and sophisticated elemental imaging and quantitation. Taken together, we described the molecular basis of negative feedback by inhibin in the ovary, engineered *ex vivo* environments for ovarian follicle development, and discovered the first external signal of a new organism, the zinc spark. Some of this work is being applied to patients who are in danger of losing their fertility in a variety of settings, including during cancer care, a medical field called oncofertility. Advances in reproductive science beckon us to see ourselves in the future, and with the discoveries described here, our view extends further.

## Graphical abstract

**Figure:**
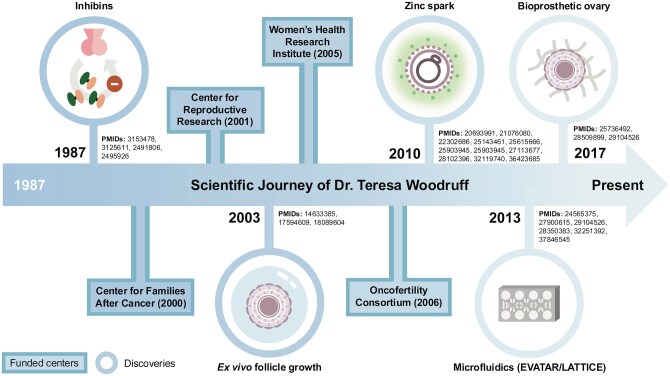
My career began in 1985 with admission to graduate school at Northwestern University, followed by a postdoc at Genentech and a faculty position at Northwestern University in 1995. Along the way, we made key discoveries and created centers that benefited the field.

## In the beginning

**Figure 1.**
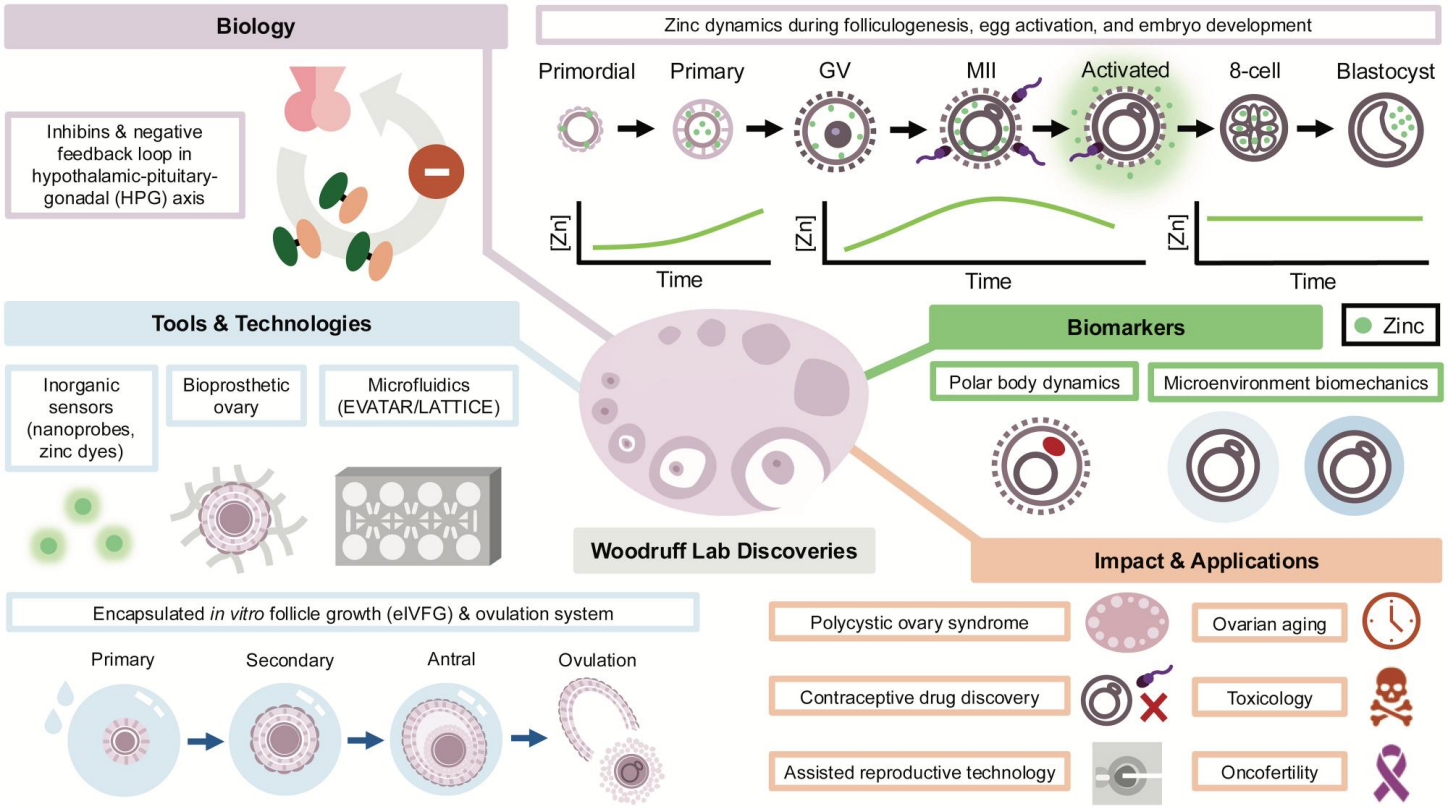
**Our work spans endocrine systems, ovarian biology to oocyte development, chemical probes to microfluidic hardware, new fields of medicine (oncofertility), and the next generation of IVF markers**. My career began with admission to graduate school in the fall of 1985 and acceptance into the laboratory of Kelly Mayo in 1986. My first project was to clone the subunits of inhibin, and it was during this molecular work that Kelly introduced me to Neena Schwartz, where we studied the negative feedback loops driving reproductive cyclicity. My group made a number of key advances, including the growth of the ovarian follicle *ex vivo* with Lonnie Shea (encapsulated *in vitro* follicle growth), the discovery of the Zinc Spark with Tom O’Halloran, the development of the EVATAR microfluidic system with Julie Kim and Joanna Burdette, and the creation of a bioprosthetic ovary with Monica Laronda. These advances included a set of tools and technologies, as well as biomarkers that are being used to uncover more about the reproductive system and systems biology, in the case of the EVATAR/LATTICE. In the end, the promise of fundamental science in medicine is that tomorrow’s patients will be treated better than today’s. And in so doing, we hope that the infertility issues faced by PCOS patients, those with advanced ovarian aging or reproductive dysfunction, either idiopathic or drug- or radiation-induced, can be mitigated, and patients can have lives with the family size or not, of their choosing. GV, germinal vesicle; MII, meiosis II; Zn, zinc.

When asked to contribute to MHR’s new Legacy Series, which reviews key discoveries made over a career (see Fig. 1), I hoped that the effort would highlight the extraordinary work of my trainees and inspire newcomers to join the field and create the next generation of discoveries that help us understand reproductive persistence. This work was done in a field of science that is small (Duncan *et al*., 2014) and collegial, and the best advances are those made collaboratively (Ataman *et al*., 2018). Our lab originally focused on the female reproductive system and began with the race to sequence the inhibin genes in the days before the Human Genome Project was funded. There is debate about the value of the $3B price tag for that global effort, but from the vantage point of the one in the trenches, the automating of sequencing hastened my discoveries by allowing us to focus on the biology rather than base pairs. Sequencing the subunits of inhibin (α and β_A_) provided us with a publication, but more importantly, the means to discover how the ovarian follicle controlled the reproductive cycle (Woodruff *et al*. 1987, 1988, 1989; D’Agostino *et al*., 1989). More details can be found elsewhere (Woodruff and Mayo, 1990; Cook *et al*., 2004; Lerch *et al*., 2007; Makanji *et al*., 2014; Woodruff, 2019), but briefly, the knowledge gap at the time was the molecular basis of the reproductive negative feedback pathway. Inhibin is a dimer of an α- and one of two β-subunits (β_A_ and β_B_). Using *in situ* hybridization, the precise regulation of the inhibin subunits in response to FSH was measured in ovarian follicles during an estrous cycle (Woodruff *et al*., 1987, 1988, 1989; D’Agostino *et al*., 1989) and correlated to the phase of the reproductive cycle in mouse, human, and then other species (Kenny and Woodruff, 2006). The subunits of these dimeric hormones are discordantly expressed and are controlled by FSH in the follicular phase and LH at ovulation. In newly selected follicles, inhibin α- and β_B_-subunits are expressed, with the α-subunit always in 20× excess to the beta-subunit to drive inhibin assembly versus activin. Similarly, inhibin B is the dominant negative feedback hormone of the early follicular phase, prior to appreciable estradiol and inhibin A levels. Once Graafian (mouse) or dominant (human) follicles are selected, the β_B_-subunit is downregulated in favor of β_A_ expression, leading to the production of inhibin A. It is not known what downregulates β_B_-subunit expression, while the α- and β_A_-subunits are profoundly downregulated by LH and supraphysiological FSH.

It is beyond the scope of this short review to explicate the mechanism of inhibin action in the pituitary, but briefly, FSH subunit expression is under the positive, constitutive control of activin B, which is expressed in the gonadotrope cell (Bernard *et al*., 2001; Thompson *et al*., 2004). Receptor complexes on the gonadotrope have inhibin B and inhibin A specificity and have been elegantly detailed by Dan Bernard and Tom Thompson, both former postdocs (Thompson *et al*., 2003, 2005). Inhibin B requires transforming growth factor (TGF)β receptor type III-like (TGFBRL3) in complex with betaglycan and activin RII. Based on the physiological evidence, inhibin B is in equilibrium with the receptors in young animals, and when circulating levels drop by just 10%, negative feedback is relieved proportionally. Inhibin A functions through the activin type II receptor and the TGFβ type III receptor (also known as betaglycan). The switching of ligand and receptors over the course of 14 h in the mouse pituitary and 5 days in the human menstrual cycle creates complexity, but also ensures a higher FSH pressure is created when needed to ensure limiting FSH during the cycle. The follicles with the highest level of FSH receptor progress, while those with lower levels undergo apoptosis. This process can be circumvented by exogenous gonadotropins, for example in IVF cycles, but to ensure the precise selection of a species-specific number of follicles in natural cycles, inhibin B and then inhibin A from the ovary create the molecular circumstances for FSH regulation that ensures cyclicity.

Many important facts emerged from the molecular, structural, and physiological studies. First, as described above, despite the remarkable β-subunit ‘switching’ between beta-subunits, the overall ‘inhibin tone’ on FSH negative feedback is not altered during an individual follicle phase (Woodruff *et al*., 1996). Second, inhibin B is a good marker of the newly growing follicles in each cycle, while the inhibin A level absolutely correlates with estradiol and is the inhibin species present at ovulation. The differential expression of inhibin becomes germane as the animal or individual approaches reproductive senescence when the number of recruitable follicles declines. Fundamentally, as the ovarian reserve diminishes, the levels of inhibin B fall slightly, allowing FSH levels to increase in a similarly marginal way. This relief of inhibin B negative feedback thus creates an FSH pressure to ensure the increasingly fewer remaining follicles are brought to an ovulatory cycle. The commensurate lowering of inhibin B and elevation of FSH provide the earliest signals of an advancing menopause. Once all follicles are depleted, FSH is no longer under the negative feedback control of inhibin B or inhibin A, and the pituitary hormone increases to its postmenopausal level.

Negative feedback is a classic endocrine pathway, generally set at homeostatic levels at the time of development. This includes the male reproductive system, which also relies on inhibin to negatively regulate FSH after puberty. The female reproductive system co-opted the negative feedback signaling pathways in a complex and resilient way to enable the necessary plasticity of ovarian follicles that are constantly being recruited from a dormant initial pool that must give way to pregnancy of different durations across species (Woodruff *et al*., 1991), different light–dark cycles (Kenny *et al*., 2002a,b; Bristol and Woodruff, 2004), and the drive to extend reproductive cyclicity until the ovarian pool is depleted and reproductive senescence begins (Houmard *et al*., 2003). Each of these steps depends on the molecular precision of FSH regulating the inhibin subunits in specific follicle groups over time, and our lab examined each of these stages from mouse to human (D’Agostino *et al*., 1989; Klein *et al*., 2004). Uncovering these mechanisms ushered in newer studies on the structure, receptors, and signaling pathways in the pituitary that toggle the inhibin signal centrally (Bernard *et al*., 2001; Thompson *et al*., 2003, 2005). Inhibin is the singular molecule that enables the great negative feedback endocrine circuit to be precise and physiologically malleable. Inhibin is a remarkable hormone that enables the negative feedback endocrine circuit for the reproductive system to be both precise and physiologically malleable (for example, during pregnancy). When it evolved, inhibin provided the signaling complexity that drives reproductive cyclicity in all animals (Zhu *et al*., 2010). My trainees are continuing to unravel the signaling pathways that drive this feedback system and are opening new targets for endocrine drugs that could assist those with infertility and cancers (Xu *et al*., 2022; Kappes *et al*., 2023; Ongaro *et al*., 2025).

## Diving deeper

The negative feedback cycle led to the question of ovarian follicle selection over time and the eventual isolation and culture of individual follicles with the development of an *in vitro* culture system that recapitulated all the hormone patterns of inhibin even in an *ex vivo* setting (Skory *et al*., 2015; Xiao *et al*., 2017). Underlying this question was the establishment of the original ovarian follicle pool at birth, representing 10 000 primordial follicles in the mouse and 1 million primordial follicles in the human. The primordial follicle is fascinating because it represents the reproductive potential of the female and must be managed over the reproductive lifetime of the organism. Growing the follicle outside the body was difficult to achieve and was first accomplished by John Eppig, leading to the birth of Egbert, the mouse named for its origin from a primordial follicle (O’Brien *et al*., 2003). This work relied on the isolation of follicles and culture in a dish, followed by fertilization and a live birth and was based on the pioneering work of Roger Gosden (Picton and Gosden, 2000). Building on these studies, we elected to take a bioengineered approach, speculating that high-fidelity, reproducible ovarian follicle development with oocyte growth requires precise 3D interactions between the egg and surrounding somatic cells to maintain its energy and signaling pathways.

These studies, done in collaboration with Lonnie Shea and his lab, resulted in ovarian follicle development that now spans all follicle stages of development and from mouse to human (more on the latter will follow later) (Xu *et al*., 2006, 2009a,b,c; Hornick *et al*., 2013; Laronda *et al*., 2014; Kniazeva *et al*., 2015; Xiao *et al*., 2015a,b).

Of the various discoveries we made with the *in vitro* follicle growth and oocyte maturation system (collectively described as encapsulated *in vitro* follicle growth, or eIVFG), one of the most profound was our ability to toggle the endocrinology of the cultured follicles simply by changing the rigidity of the surrounding biomaterial (Pangas *et al*., 2003). The biomaterial that Lonnie selected for these studies is alginate. As a product of algae, alginate has properties that allow it to be gelled and de-gelled under physiological conditions and allow oxygen and hormones like FSH to freely diffuse in and CO_2_ and estradiol to move out. Stephanie Pangas, a graduate student at the time, was the first to test whether ovarian follicles could be cultured in an alginate bead. Not having any clear boundary conditions, Stephanie set up what we later learned was the only condition under which follicles would grow, namely, a very loose, not rigid, gel. Indeed, the first follicles were cultured in what I described as a ‘shmoo’ because the gelatinous alginate was somewhat blobby—but the follicle grew and grew. Min Xu, Pam Kreeger, and other students took on the remainder of the early studies, and we learned a great deal from those follicle cultures, notably that the follicle has on board most of what it needs—it can develop its own theca cell layer and needs no more than FSH to initiate and sustain steroidogenesis. In addition, we learned that this does not happen outside of a structured environment—e.g. sitting on flat plastic. Moreover, we learned that had Stephanie made a structurally more rigid bead in which to grow the first ovarian follicles, we likely would not have continued the nascent collaboration with Lonnie. Indeed, we went on to show that changes in the rigidity altered follicle development and hormone production, with more rigid environments leading to androgen production over estrogen and smaller follicle development, with theca dominating the granulosa cells’ ability to convert precursors to estradiol. The follicles were not dying; they just were not developing. This physiology was very much like the common ovarian pathology called polycystic ovarian syndrome (PCOS), where follicles grow to the small antral stage and then stop further development, making excess androgen. The infertility and the masculinizing hormones are both clinically significant, and this work was the first to link the clinical manifestation of rigid ovaries with follicle function (West *et al*., 2007; Woodruff and Shea, 2007). The biology of the stroma—an understudied and underappreciated part of the ovary—then became central to the ongoing work of Francesca Duncan, who has studied the stroma from the perspective of ovarian aging, and Monica Laronda, who has continued the work on ovarian reconstruction for pediatric patients—two ends of critically important spectrums of development (Amargant *et al*., 2025; Balough *et al*., 2024; McDowell *et al*., 2024; Hughes *et al*., 2025). In the meantime, creating the endocrine conditions of a human pathology suggested we had a robust system for continued study, which allows for deeper and richer evaluation methodologies and new discoveries (Chen *et al*., 2022; Zhang *et al*., 2023; Zaniker *et al*., 2024).

I want to return for a moment to the human follicle eIVFG cultures, given their potential utility to human infertility. Groups working around the world created *in vitro* environments for follicle development, including Telfer and Schmitz (Herta *et al*., 2018; McLaughlin *et al*., 2018) and many others. The contributions that Lonnie’s and my groups made included the proof of principle, which ushered in further study of the mechanisms of follicle activation, development, and identity of potential contraceptive pathways. The first human eggs matured from the ovarian follicles were few, given the paucity of human tissues that could be used in the development of the technology, but they were definitively the first human eggs matured outside the body and remain a milestone in reproductive biology. The first confirmed *ex vivo* human follicle growth with appropriate signatures of egg development was done by Shuo Xiao, a postdoc working with me at the time. This groundbreaking result suggests that human follicles can reach a stage where they produce meiotically competent oocytes. Hindering further work in this area continued to be the lack of funding for reproductive science and the availability of tissues. These represent the bottleneck in reaching the goals of ovarian follicle development for the purpose of providing restoration of fertility for young cancer patients, which must be addressed by the next generation of investigators. The translation of this global work was the development of a new field of medicine, oncofertility (Woodruff, 2013, 2015; Ataman *et al*., 2020; Salama *et al*., 2020; Woodruff *et al*., 2021; Ataman *et al*., 2022). The notion that fundamental and applied reproductive research could enable new options for cancer patients was critically important when the early work was being done, as few options were available to patients. There is good news for many patients using existing assisted reproductive technologies, and work is ongoing to develop options that are appropriate for more individuals. Moreover, we took it upon ourselves to work through the legal and ethical issues surrounding this topic, much of which was summarized in one of six books written on the topic of oncofertility, *Oncofertility: Ethical, Legal, Social, and Medical Perspectives* (Dolin *et al*., 2010; Woodruff *et al*., 2010), and the promise of fundamental science in medicine is that tomorrow’s patients will be treated better than today’s, and many labs are continuing this work around the globe. Furthermore, it is exciting to see the many advances made by my students using this technology and building the next generation of tools that will supersede our original discoveries, capitalizing on the maxim and promise of that original scientific work (Zaniker *et al*., 2024; Brunette *et al*., 2026; Pattarawat *et al*., 2025).

## What makes a good egg?

This question was the driving animation of a series of postdocs in my lab who made successively critical discoveries, followed by a true cataclysmic discovery that changed everything we know about egg biology. As an endocrinologist, I typically looked at the follicle unit from the perspective of the somatic cells—the granulosa and theca cells that were responsible for hormone production. But to really understand the potential of the *in vitro* follicle work that Lonnie and I were doing, we needed to be able to assess the oocyte and signatures of maturation that would lead to a healthy live birth and further development. Our question was whether the maturation of the egg, when done in the context of the follicle, would provide better metabolic regulation during times when the growing oocyte is dependent on the somatic cells for metabolic precursors. However, a key constraint for this work was the determination of genetic, metabolic, and developmental health that could be assessed without destroying the egg. We began with an evaluation of the polar body, the asymmetrically cleaved cell that contains half of the DNA and a small amount of cytoplasm. Zexu Jiao began to meticulously isolate the first and second polar bodies and determined the transcript levels in these ‘waste cans’ of reductive division (Jiao *et al*., 2012; Jiao and Woodruff, 2013a,b; Jiao *et al*., 2014). She assessed the effect of biological age and responses to the local microenvironment. Age-related changes in oocyte quality are well documented, and the *in vitro* environment mimics, in some respects, this physiology. Jiao showed that there was a corresponding decrease in key metabolic transcripts with age. At the time, we were completely fascinated by the endocrinology of the follicles grown under rigid and permissive conditions and applied the polar body analysis to follicles developed with these parameters. We were amazed to see that the external environment relayed specific information to not just the follicle but also to the egg that could be read out in the transcriptome of the matured egg. This evidence further implicated a holistic approach to the study of fertility restoration, which we ultimately did with the ovarian bioprosthetic. Returning to egg quality markers, the fate of eggs cultured under more and less rigid conditions also mimics what happens during ovarian aging, and so we began a critical collaboration with John Marko, an extraordinary biophysicist, and graduate student, Alison Kim, originally, who was later joined by two exceptional postdocs, Francesca Duncan and Jessica Hornick. John had shown that the physical dynamics of mitotic chromosomes could be measured by drawing out a single chromosome filament and placing it between a holding and a measuring pipette, applying force, and determining elasticity. Alison was a fearless graduate student, and she set up the first meeting with John, where we discussed the fact that meiotic chromosomes had not been studied. Given our recent work on the egg outcomes based on alginate rigidity and the human models of PCOS and aging, we developed a hypothesis and a grant to apply this technology to meiosis. The key contributor to the remarkable outcome of this collaboration was Jessica Hornick, a scientist with incredibly gifted hands who was able to pull chromosomes out of the meiotic spindle and place individual threads on the Marko instrument. Francesca was intellectually leading in egg biology with Richard Schultz, and she and Jessica formed an incredible team (Alison graduated from the lab by the time this collaboration got fully energized), and the discoveries made stand as among the most remarkable (Hornick *et al*., 2015). First, meiotic chromosomes are 2-fold more rigid than mitotic chromosomes—why, we still don’t know—and chromosomes isolated from reproductively aged animals are 2.7-fold more rigid than their young counterparts. Structure and function continue to be hand-in-hand in ways we had never understood before. The follicle structure underlies its function as an endocrine unit, and the oocyte depends on this structure for its transcriptional and chromosomal maturation and physical health. Eureka moments all around. Despite the brilliance of this study, the new discoveries regarding polar body transcriptomics would not allow us to easily and routinely determine if an egg matured in the eIVFG setting would be of sufficient quality to transfer to a patient seeking fertility options. Instead, that non-invasive tool was a serendipitous discovery that was unanticipated by the field for its importance to the biology of meiosis and, more importantly, placed the main character, zinc, in a wholly new realm as a signal transduction factor on par with calcium and phosphate.

## The zinc spark

To tell this story is to seem somewhat apocryphal, but it is true. My husband, Thomas O’Halloran, is a chemist, and I’m a reproductive scientist, and one morning, as we were walking along the beach of Lake Michigan near our home in Chicago, he asked me why sperm had so much zinc, and I said the three most unfortunate words of my life: ‘I don’t care’. With further prodding, I explained that I didn’t care about sperm, but if there was something interesting about zinc in the oocyte, we could talk. In the end, Alison and many members of our groups made some of the most important discoveries in egg biology. The team demonstrated that zinc is required for the completion of meiosis. Twenty billion zinc atoms are accrued during meiotic maturation to advance across a previously undescribed checkpoint that requires this influx, and at the time of fertilization, the excess zinc is released in what is known as the zinc spark (Kim *et al*., 2010, 2011; Bernhardt *et al*., 2011; Bernhardt *et al*., 2012; Kong *et al*., 2012, 2014, 2015; Que *et al*., 2015; Duncan *et al*., 2016; Zhang *et al*., 2016; Mendoza *et al*., 2017, 2022; Que *et al*., 2017, 2019; Hu *et al*., 2020; Lee *et al*., 2020; Seeler *et al*., 2021; Sue *et al*, 2022; Chen *et al*., 2023; Balough *et al*., 2026). There are many outcomes to this discovery that we have detailed in a separate review that is forthcoming, but for the purpose of this retrospective, among the truly fascinating aspects of this discovery is that no one had looked for a role for zinc in the egg before our study. Part of the paucity was because studies on female biology are always less likely than male (Tingen *et al*., 2010; Klein *et al*., 2015; Mansukhani *et al*., 2016; Prakash *et al*., 2018; Woitowich *et al*., 2019; Mulles *et al*., 2025), but it is equally true that without Tom’s incredible toolkit, including resources at Argonne National Laboratories and a series of zinc dyes (Hong *et al*., 2014; Kong *et al*., 2015; Que *et al*., 2015; Garwin *et al*., 2019), in addition to his keen insights on zinc biology, we could not have made the initial or ongoing discoveries.

The second outcome of this work is the placement of zinc alongside phosphate and calcium as the key inorganic signaling pathways that control the cell cycle. Phosphate works through covalent bonding to make and break ATP, while calcium works through fluxes across membranes as an ionic signal. In our studies of the transition between the oocyte and egg, we were able to show that zinc is a true signaling metal, not just a structural metal in zinc finger proteins, and has features of both covalent bonding and ionic fluxes. And finally, we knew that the zinc spark, an external signal of successful fertilization, a signature of life, could be the precise marker that would allow us to non-destructively assess egg readiness to proceed through development. The proof that human eggs also exhibited the zinc spark was the brilliant work of Francesca Duncan and Emily Que, and we now have evidence of the conservation of zinc fluxes from flies to primates as a necessary factor for germ cell development. Mechanistic studies and the transition to human IVF labs are underway, but the fundamental discovery stands as a singularity of something happening every reproductive cycle, but just out of biological reach. One of those biological processes that seemed difficult to unravel was the earliest activation of follicles and, in a tour de force study assessing zinc in follicle activation using five different physical methods, Yu-Ying Chen showed a dynamic pattern of zinc uptake in newly activated primordial follicle oocytes. This observation may be meaningful for the necessary transcriptional activation as the largely dormant primordial oocyte begins transcription and growth, and we speculate that it may also be part of the initiation signal that has long eluded us (Chen *et al*., 2023). The zinc spark has shed light on a new domain of research, and it is exciting to see what will come next.

## Cycling back to endocrinology

Hallmarks of my lab and its discoveries have been to embrace or create new technologies and the value placed on interdisciplinarity. These principles are born out in the work we did to create an *in vitro* mimic of the reproductive system (EVATAR and LATTICE, described below) and the follow-on work to create an ovarian bioprosthetic. Microfluidics might seem the furthest afield, but it was a natural output of having ovarian follicle development that could be controlled *in vitro*. This led to the question of whether we could drive a full reproductive cycle in a dish, but that required creating a system to support that biology. I have always believed that the breakthroughs in cell biology and the eventual development of drugs used to treat diseases that originate from these cells are tightly intertwined with the discovery of technologies that advanced our underlying knowledge of them. Indeed, the origin story of the cell is the observation of cork and then sperm in the first microscopes of Van Leeuwenhoek. Arguably, the next leap forward was made in the lab of Robert Koch when his technician, Julius Petri, invented the Petri dish for culturing bacteria. This simple circular dish with a lid, which allows for gas transport while simultaneously limiting contamination, was a game changer and has been used unmodified for nearly 140 years.

Since that time, technologies ranging from centrifuges to single-cell genomics have entered the cell biology toolkit, but the Petri dish has been unaltered. Indeed, the Petri dish, which began as a tool on which to culture bacteria, was adopted to the culture of all cell types, including those that ordinarily exist in multidimensional compound tissues that sense and respond to cells from adjacent or distant organs, such as the ovarian follicle. What began as a method to culture bacteria was adopted to the culture of all cell types, including those that ordinarily exist in multidimensional compound tissues that sense and respond to cells from adjacent or distant organs, like the ovarian follicle. Moreover, cells grown in Petri dishes are cultured in static conditions that accumulate waste products and are removed only when the media is replenished. Thus, the general biology being studied is under the persistent pressure of metabolic waste. Working together with Draper Labs in Cambridge, we first developed a system called EVATAR and, more recently, LATTICE, which allows for fluid exchange under precise microfluidic control and single-, double-, or multiple tissues to be connected, providing the feedback and feedforward that ordinary endocrine systems have *in vivo* (Laronda *et al*., 2013; Jakus *et al*., 2017; Laronda *et al*., 2017; Xiao *et al*., 2017; Gargus *et al*., 2020; Campo *et al*., 2023). While still under development, I feel these microfluidic systems are going to have a profound impact on the field of endocrinology and allow new discoveries that can only be made when tissues talk to each other, will rewrite many signaling pathways that have been determined in supra-non-physiological conditions, and will allow new and personalized drug development that is unavailable today. Our contribution to the biological proof of principle was the connection of ovarian follicles or whole ovaries connected microfluidically to human oviduct, endometrium, cervix explants, and liver organoids. Using this configuration, we generated 28-day reproductive cycles in a dish with hallmarks of each tissue functioning throughout and differentially to the rising and falling estradiol, followed by progesterone. We also demonstrated endocrine fidelity to hormone signaling by clamping hCG, resulting in an extended luteal phase, identical to the *in vivo* condition.

## Ovarian bioprosthesis

The need to develop alternative fertility restoration technologies beyond individual follicle growth ushered in the next generation of discoveries in our lab, including ovarian bioprosthetics. At its best, *in vitro* follicle development allows for individual oocyte maturation and the theoretical potential for ICSI and embryo transfer. For pediatric patients, the need for full ovarian endocrine function is more pressing than fertility restoration, giving rise to the work by Monica Laronda and others in the lab and with Ramille Shah, a biomedical engineer, to create ovarian bioprosthetics (Laronda *et al*., 2015; Jakus *et al*., 2017; Laronda *et al*., 2017). Using 3D printing principles, we developed scaffolds onto which ovarian follicles could be placed and give rise to development and ovulation *in vitro*, in EVATAR, and *in vivo*. Whether this kind of tissue mimetic can be used for human utility is under investigation in several labs and is at the cutting edge of this field of work.

## And in the end

So, what do we know now that we didn’t know when we began these studies? First, that inhibin B, then inhibin A, create a sequential inhibin tone maintaining the negative feedback pressure of growing follicles, followed by a dominant follicle. We also know that the ovarian follicle is an autonomous unit, having everything onboard that it needs for growth, oocyte development, steroidogenesis, ovulation, and luteal transition, providing it has the right physical environment—describing for the first time a necessary role for the stroma in follicle development. We also know now that zinc is a signaling molecule, not just a structural feature of zinc finger proteins, and is necessary to the transition out of anaphase into meiosis II, and then must be exported at the time of fertilization to progress through development. And finally, we know now that all this work must be translated to human use. While we have developed ovarian follicle maturation strategies, the ovarian bioprosthetic, and even a field of medicine named oncofertility, progress has been slower in meeting patient needs. This is motivating to the ongoing work of many in the field, and I hope I passed that baton on successfully some years ago as we developed the Oncofertility 2030 plan and empowered a new generation of leaders to take the mantle, the grants, and the leadership necessary to enable endocrine and reproductive health for cancer survivors.

The hallmarks of my work have included asking the right questions, having brilliant students, working with great interdisciplinary collaborators, embracing or building the technologies needed to solve the problems, and always being ready to be taught. I hope that I have contributed to the good of the field, the expansion of knowledge, and the enabling of better health for patients in the future. For all this work, I thank my trainees and the field. There was much we did not know, but now we do, and I hope the legacy of this work is judged as good science.

## Author Biography

**Teresa K. Woodruff, PhD,** is a leader in higher education and an internationally recognized biologist specializing in reproductive science. She is president emerita of Michigan State University (MSU), where she also served as provost and executive vice president for academic affairs. In recognition of her leadership at MSU, she was named Distinguished Woman in Higher Education Leadership and a Newsmaker of the Year by *Crain’s Detroit Business*.  Dr. Woodruff graduated *summa cum laude* from Olivet Nazarene University, where she received the Maggie Sloan Crawford Award, the highest honor conferred by the faculty, and the ‘O’ Award, the institution’s highest distinction for alumni. She earned her PhD from Northwestern University and was later recognized with the university’s highest alumni award, as well as an award from the Alumnae of Northwestern University.  Following postdoctoral training at Genentech, Dr. Woodruff returned to Northwestern University, where she served as the Thomas J. Watkins Professor of Obstetrics and Gynecology and Dean of the Graduate School. She is widely recognized for her dedication to teaching and mentoring and was awarded the Presidential Award for Excellence in Science, Mathematics, and Engineering Mentoring by President Barack Obama in an Oval Office ceremony. For her scientific accomplishments, Dr Woodruff was honored by President Joseph R. Biden Jr with the National Medal of Science. She holds two honorary Doctor of Science degrees, from Bates College in Lewiston, Maine, and the University of Birmingham School of Medicine in Birmingham, UK. She is also featured in *Wonder Women of Science: How 12 Geniuses Are Rocking Science, Technology, and the World*.
